# Comparative proteomics analysis provide novel insight into laminitis in Chinese Holstein cows

**DOI:** 10.1186/s12917-015-0474-x

**Published:** 2015-07-23

**Authors:** Shu-Wei Dong, Shi-Dong Zhang, Dong-Sheng Wang, Hui Wang, Xiao-Fei Shang, Ping Yan, Zuo-Ting Yan, Zhi-Qiang Yang

**Affiliations:** Key Laboratory of Veterinary Pharmaceutical Development of Ministry of Agriculture/ Engineering & Technology Research Center of Traditional Chinese Veterinary Medicine of Gansu Province/Lanzhou Institute of Husbandry and Pharmaceutical Sciences of Chinese Academy of Agricultural Sciences, Lanzhou, 730050 People’s Republic of China; Key Laboratory of Yak Breeding Engineering, Lanzhou, 730050 People’s Republic of China

**Keywords:** Comparative proteomics, 2-DE, Laminitis, Plasma, Dairy cow

## Abstract

**Background:**

Laminitis is considered as the most important cause of hoof lameness in dairy cows, which causes abundant economic losses in husbandry. Through intense efforts in past decades, the etiology of laminitis is preliminarily considered to be subacute ruminal acidosis; however, the pathogenesis of laminitis needs further research. The differentially expressed proteins (DEP) were detected in plasma of healthy cows and clinical laminitis cows by two-dimensional gel electrophoresis (2-DE) and identified by matrix-assisted laser desorption/ionization time-of-flight mass spectrometry.

**Results:**

Nineteen protein spots were differentially expressed, and 16 kinds of proteins were identified after peptide mass fingerprint search and bioinformatics analysis. Of these, 12 proteins were differentially up-regulated and 4 down-regulated. Overall, these differential proteins were involved in carbohydrate metabolism, lipids metabolism, molecular transport, immune regulation, inflammatory response, oxidative stress and so on.

**Conclusions:**

The DEPs were closely related to the occurrence and development of laminitis and the lipid metabolic disturbance may be a new pathway to cause laminitis in dairy cows. The results provide the theory foundation for further revealing the mechanism of laminitis and screening the early diagnostic proteins and therapeutic target.

## Background

With China’s steady and high-speed economic development, the import amount of dairy cow, frozen sperm, and embryo are strongly increasing for meeting the dairy industry demands. At the same time, infectious diseases have been effectively controlled, but the animal welfare and some nutrition metabolic diseases are easy to be ignored. Lameness is an increasing disease in dairy cows that are associated with higher production, more intensive feeding, and confined conditions. It has been considered as the third serious disease, following mastitis and reproductive diseases in dairy cows in China. The problem’s prevalence is a significant contributor to compromised animal welfare and the economic loss in the dairy industry.

Laminitis—an inflammation of the laminar corium in the hoof wall—is a common underlying cause of lameness, accounting for 41 % of the cases of lameness identified in cows [1]. It can result in many kinds of claw horn lesions including sole hemorrhages, white line disease, and sole ulcers in dairy cattle [2], which highly affect a cow’s welfare, production, and longevity. Laminitis is divided into two stages depending on clinical presentation: subclinical and clinical stages. The subclinical phase usually does not have any obvious evident syndrome, so the sick cattle is always ignored and misses the best opportunity of the treatment. Thus, laminitis has been an invisible killer of dairy cows. In recent years, many scholars have studied the etiology of laminitis [3–5], laminar morphology [6], metabolism [7], pathophysiology [1], clinical diagnosis [8, 9], and comprehensive treatment [10] in laminitis cows. Through intense efforts to understand the root cause of clinical laminitis in past decades, the etiology of laminitis is preliminarily considered to be subacute ruminal acidosis (SARA) [11, 12], but the pathogenesis of laminitis is not clearly understood. Laminitis that was been proved refractory to conventional research approaches has been especially frustrating for clinicians because of the dearth of new treatment strategies for age-old diseases [13].

Proteins are the final executants of biological functions in life. Tiny alterations of protein expression happen in all physiological and pathological processes. Thousands of proteins are secreted in plasma by cells or tissues that contain rich information concerning overall pathophysiology of the patient or diseased animal [14]. Therefore, the analysis of the profile of plasma protein alterations is a promising way to find the potential biomarker and shed light on the pathogenesis of disease. At present, comparative proteomics have identified a large number of differentially expressed proteins associated with diseases. Although roles and mechanisms of such proteins in the pathogenesis need to be further proved, at least some of them may be potential biomarkers for early diagnosing diseases. So, the technology based on proteomics has a wide prospect in clinical diagnosis and prevention of diseases.

However, the proteomics application in animal science and veterinary is still in its infancy, and studies have mainly been directed toward production traits [15] and epidemic diseases [16, 17]. Proteomics also provides a novel approach to investigate the pathogenesis of laminitis [18]. Hannah analyzed the proteomics of lamellar tissue of equine laminitis and discovered that keratins could serve as serum biomarkers for the developmental phase of endocrinopathic laminitis [19]. So far, only two proteomic studies on claw tissues in dairy cattle have been published [20, 21], and very little information are available on the presence of specific proteins in plasma. Laminitis is a local manifestation of systemic metabolism disorder in cows; therefore, some corresponding changes may occur in plasma proteome [22].To investigate this broad hypothesis, two-dimensional gel electrophoresis (2-DE) coupled with matrix-assisted laser desorption/ionization time-of-flight mass spectrometry (MALDI-TOF MS) was used to detect differentially expressed proteins in plasma, as a first step toward characterizing global alterations in dairy cows with clinical laminitis. The objective of our study was to screen the potential protein biomarkers that monitor the occurrence and the drug target of laminitis and shed light on pathogenesis with the intent of developing novel preventive strategies and therapeutic approaches. The current study provides a novel report of comparing proteomics analysis of plasma in dairy cows with laminitis.

## Methods

### Ethics statement

The experiment was conducted in accordance with the good animal practices requirements of the Animal Ethics Procedures and Guidelines of the People’s Republic of China. This study was approved by the Institutional Animal Care and Use Committee of Lanzhou Institute of Husbandry and Pharmaceutical Sciences of CAAS (Approval No. LIHPSACUC2011-012).

### Case definition of laminitis

A total of 4680 Holstein cows were selected from May to August in 2011in the Reproduction and Breeding Demonstration Center of Chinese Holstein Dairy Cow of Gansu Province, which was the member of the Chinese National technological system of dairy industry. Laminitis was identified with at least one of the following claw horn lesions: double sole, solar ulcer, solar hemorrhage, white line disease, and solar abscess [23]. A case of cow usually has the following clinical signs: redness, heat, pain, or sensitivity to percussion, and swelling of the lamellar hoof, as well as the corresponding systemic symptoms, which are diagnosed by hoof trimmers and veterinarians. A total of 36 adult cows in lactation with acute laminitis were selected as a sick group from the dairy herds during the routine herd trimming, with at least one hoof suffering from laminitis. A total of 15 healthy dairy cows with no evident clinical signs of other diseases were included as a control group. All of the cows enrolled in the study aged 3–5 years, were around 400 kg in body weight, and had not received any drug treatment for 1 month before trials. All procedures were followed up by a veterinary assistance and according to the corresponding ethical and animal welfare guidelines.

### Collection and selection of blood samples

Blood samples were withdrawn from the jugular vein by cava venepuncture using a 16-gauge needle in 10 mL tubes and immediately transferred into sealed vacutainer glass tubes that contained EDTA-K2 as an anticoagulant (for plasma). After collection, samples were placed on ice and transferred to the laboratory in 4 h. Plasma was obtained by centrifugation at 2000 *g* for 15 min at 4 °C. To emphasize proteomic differences between the groups while eliminating potential individual contributions, six cows were randomly selected from the same group and equal volumes of plasma were pooled from them. Two mixed plasma samples were available for the next proteomic experiment, which included comparison between sick and healthy groups.

### Preparation of protein samples and depletion of abundant proteins

Plasma samples were analyzed using two commercial kits: Albumin and IgG Removal kit (GE Healthcare, NJ USA) and 2-D Clean Up kit (GE Healthcare, NJ USA). The most abundant proteins of albumin and immunoglobulin (IgG) in plasma were removed by the immune affinity-based method according to the manufacturer’s instructions. After fractionation, samples were desalted using the 2-D Clean Up kit.

The protein precipitate in samples were resuspended in a lysis buffer containing 8 M urea, 2 % 3-[(3-cholamidopropyl) dimethylammonio]-1-propanesulfonate (CHAPS) (w/v), 18 mM dithiothreitol (DTT), 1 % ampholytes (v/v), and bromophenol blue (silver-stained gels) or 30 mM Tris-HCl, and sonicated to completely dissolve the aggregated protein (15 s, 0.5 cycles/s, 60 % amplitude). Samples were centrifuged at 16,000 g for 15 min at 4 °C in a 5415R centrifugal machine (Eppendorf 5415R, Germany) to get suspensions, which were diluted in the lysis buffer using protease inhibitor (Complete EDTA-free Protease Inhibitor Cocktail Tablets, Roche, Spain) until the final protein concentration was 5–10 μg/μL as determined using the 2-D QUANT KIT (GE Healthcare, NJ, USA). Samples were aliquoted once the experiment was over and stored at −80 °C until 2-DE analysis.

### 2-DE procedure

To discover the potential new biomarkers in plasma, 2-DE was performed to screen differentially expressed proteins between healthy and sick cows. Isoelectric focusing (IEF) was run on an Ettan IPG phor II (GE Healthcare, CA, USA) using 24 cm nonlinear immobilized pH gradient strips (pH 3–10; GE Healthcare). Protein samples (150 μg) pooled with rehydration solution (8 M urea, 2 % CHAPS, 20 mM DTT, 0.5 % (v/v) immobilized pH gradient (IPG) buffer (pH 3–10), and 0.001 % bromophenol blue) were placed for 12 h at 4 °C. The linear ramping mode of the IEF voltage was applied in the focusing program as description in reference [24]. Strips were sequentially incubated for 15 min in 10 mL equilibration solution with 2.5 % (w/v) DTT or iodoacetamide (IAA). Second-dimension electrophoresis was performed on 12.5 % sodium dodecyl sulfate gels in an Ettan DALT six apparatus (Amersham Bioscience, Uppsala, Sweden) with constant power at 5 W per gel for the first 30 min, and then at 12 W per gel for 6–7 h until the bromophenol blue line reached the bottom of the gels. Gels were treated in triplicate and silver stained according to published procedures [25]. Gels were scanned at 300 dpi resolution (UMAX USB2100XL, Taiwan, China), and the profiles were renamed as the experiment.

### Image analysis and protein identification

Differential analysis was performed using Image Master 2D platinum software (Version 5.0, GE Healthcare, CA, USA) for spot detection, quantification, matching, and comparative and statistical analyses. Data were averaged from three independent gels, and the mean and standard deviations were calculated and assessed for statistical significance by normalized intensities of spots. Finally the differentially expressed proteins were defined between the sick and healthy groups with a paired *t* test if *P* values were less than 0.05, and the average spot intensity was greater than threefold.

Protein spots of interest were excised manually from the gel, subjected to destaining and trypsin digestion according to the protocol described by Wu [24], and purified using ZipTip microliter plates (Millipore). MALDI-TOF MS analysis of tryptic peptides was performed on an Ultraflex TOF/TOF instrument (Bruker Daltonics). Proteins were identified by peptide mass fingerprint (PMF) using the Mascot search engine (http://www.matrixscience.com; Matrix Science Ltd., London, UK) and the Swiss-Prot 55.4 database.

### Bioinformatics analysis

The identified proteins were searched in the Uniprot database (http://www.uniprot.org/), AgBase (http://www.agbase.msstate.edu/), and published literature for their functions [26]. According to the combined search results, these proteins were divided into different functional groups. Categorical annotation was supplied in the form of gene ontology (GO) biological process (BP), molecular function (MF), cellular component (CC), as well as participation in a Kyoto Encyclopedia of Genes and Genomes (KEGG)pathway and membership in a protein complex as defined by the comprehensive resource of mammalian protein complexes (CORUM) [27].

### Validation of differentially expressed protein

To add confidence to the results obtained by 2-DE, 2 of 16 differentially expressed proteins in plasma were measured in healthy and sick groups. The concentrations of haptoglobin were detected by Bovine ELISA kits (Shanghai Institute of Biological enzyme-linked, Shanghai, China). ApoA-I in plasma was detected by immunoturbidimetric method in the automatic biochemical analyzer (Mindary 420, Shenzhen, China) using commercial test kits for human (Mindary, Shenzhen, China). The standard curve was developed with the known ApoA-I concentration (0 g/L, 0.180 g/L, 0.530 g/L, 1.26 g/L, and 2.45 g/L). The procedure was performed according to the manufacturer’s instructions.

Plasma samples (1:2000 dilutions) were added in duplicate to each well in enzyme-linked immunosorbent assay (ELISA) plate precoated with monoclonal antibody (McAb) against bovine haptoglobin, and then 10 μL of biotin-labeled McAb and 50 μL of streptavidin–HRP conjugates were added to the wells. After incubation at 37 °C for 1 h, the ELISA plate was washed for three times using PBST [0.5 % (v/v) Tween-20, PBS, pH 7.4]. Coloration was developed by 3′, 5, 5′-tetramethylbenzidine solution, and the reaction was stopped with 50 μL of 2 M H_2_SO_4_. The absorbance was measured at a wavelength of 450 nm. In ELISA test, bovine haptoglobin solutions with known concentrations (800 mg/L, 400 mg/L, 200 mg/L, 100 mg/L, and 50 mg/L) were used to prepare a standard curve according to the ELISA procedure described by the manufacture. Haptoglobin concentration in plasma was calculated according to the sample absorbance and standard curve.

### Assay of antioxidant ability of plasma in dairy cows

Plasma samples were analyzed for the total antioxidative capacity (T-AOC), malonaldehyde (MDA), super oxygen dehydrogenases (SOD), and glutathione peroxidase (GSH-Px) using colorimetric assay kits (Nanjing Jiancheng Bioengineering Institute, Jiangsu, China), and detected by microplate reader (Spectra Max M2, Molecular Devices, CA, USA). All samples were tested in duplicate. The T-AOC concentration was determined by the reaction of phenanthroline and Fe^2+^ using spectrophotometer at 520 nm. MDA was measured by the thiobarbituric acid method. SOD activity was determined by inhibiting nitroblue tetrazolium reduction due to superoxide anion generation by a xanthine–xanthine oxidase system. The GSH-Px level was determined using the direct measurement of the remaining GSH after the enzyme-catalyzed reaction. All assay procedures were performed according to the manufacturer’s instructions.

### Statistical analysis

Statistical analysis was performed using the SPSS statistical package v 17.0. Normality of data was tested using the one-sample Kolmogorov–Smirnov test. Data were analyzed by one-way analysis of variance, and differences between group means were evaluated with the Duncan test. Differences between groups were analyzed with independent *t* test (*P < 0.05*).

## Results

### Comparative proteomic analysis of plasma samples

**Fig. 1:**
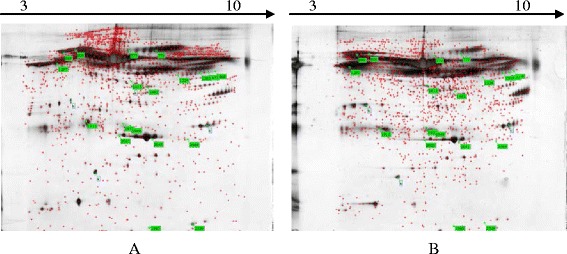
Representative image of 2-DE gel by silver stained. Note: Identified spots of differentially expression proteins are indicated by green blank with spot number. **a** is from sick cows and (**b**) is from healthy cow

**Fig. 2:**
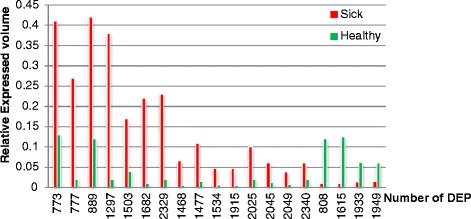
The relative expressed volume of differentially expression proteins

Scanning of 2-DE gel maps showed that several spots did not correspond to proteins, which may be due to contamination or gel impurities. Therefore, these spots were eliminated in comparative analysis. Plasma proteomic profiles of healthy and sick groups were analyzed by Image Master. Representative 2-DE profiles of healthy and sick groups are illustrated in Fig. 1a and b. Qualitative analysis revealed approximately 763 and 757 protein spots on each 2-DE images. While the quantitative analysis of the spots’ expression showed that a total of 19 stained protein spots (Fig. 1) showed significant changes of at least threefold up- or down-regulated expression in the healthy group (Fig. 2). Among them, 15 spots (M-01 to M-15) were up-regulated while other 4 spots (M-16 to M-19) were down-regulated in response to the occurrence of laminitis.

### Protein identification by MS

All differentially expressed protein spots were excised from the 2-DE gels and subjected to trypsin digestion. The peptide mixtures were submitted to MALDI-TOF MSfor identification. Peptide mass fingerprinting was searched by Mascot in NCBInr database (Version 20101030) with *other mammalian* as taxonomy. Nineteen of them were positively identified as 16 kinds of proteins (Table 1). Spot889, spot1468, and spot1477 were identified as the same protein of serum albumin, and spot2025 and spot2329 were the same protein of apolipoprotein A-IV (ApoA-IV) precursor. Finally, 4 kinds of proteins were down-regulated in expression in laminitis cows, which included ectoderm-neural cortex protein 1, glycerol-3-phosphate dehydrogenase 1-like protein, complement component 4 binding protein, and complement component C9 precursor, and 12 kinds of proteins were up-regulated in expression, which included 3-hydroxy-3-methylglutaryl-coenzyme A reductase (HMGCR), SPEG complex locus, serum albumin, complement component C9, haptoglobin, isocitrate dehydrogenase 1, 60S ribosomal protein L5, conglutinin, ApoA-IV, zinc finger protein 300-like, transmembrane protein, and apolipoprotein A-I (apoA-I). The experimental molecular mass and isoelectric point of each protein was similar to the theoretical values.

**Table 1:** Identification of differentially expressed proteins in plasma in dairy cows with laminitis by MALDI TOF MS/MS

NO. spot		Change	Protein name	NCBI accession	pI;	Mw	Coverage	Score	NO. of peptides identified
1	773	UP	3-hydroxy-3-methylglutaryl-Coenzyme A reductase(HMGCR)	gi|157785597	6.27;	99155	33 %	184	16
2	777	UP	SPEG complex locus	gi|215599780	8.83;	357571	6 %	73	15
3	889	UP	Serum albumin	gi|76445989	6.09;	55487	18 %	78	6
4	1297	UP	Complement component C9;	gi|85718632	5.66;	63327	20 %	108	9
5	1468	UP	serum albumin	gi|76445989	6.09;	55487;	18 %	73	5
6	1477	UP	serum albumin	gi|76445989	6.09;	55487	27 %	78	8
7	1503	UP	Haptoglobin, HPT	gi|94966763	7.83;	45629	47 %	136	9
8	1534	UP	Isocitrate Dehydrogenase 1, IDH1	gi|89573995	6.12;	41658	49 %	175	10
9	1682	UP	60S ribosomal protein L5, RPL5	gi|78369655	9.73;	34551	28 %	63	7
10	1915	UP	Conglutinin, CONG_BOVIN	gi|395268	5.82;	38370	27 %	73	4
11	2025	UP	Apolipoprotein A-IV precursor	gi|296480272	5.30;	42963	20 %	92	7
12	2045	UP	Zinc finger protein 300-like, ZNF300	gi|297493133	8.66;	27753	59 %	181	6
13	2049	UP	Transmembrane protein, TMP10	gi59858239	6.85;	15875	59 %	199	3
14	2329	UP	Apolipoprotein A-IV precursor, apoA-IV	gi|296480272	5.30;	42963	55 %	174	5
15	2340	UP	Apolipoprotein A-I, apoA-I, apoA-I	gi|245563	5.57;	28415	67 %	242	7
16	808	Down	Ectoderm-neural cortex protein 1, ENC1	gi|118151210	6.40;	67257	46 %	176	15
17	1615	Down	Glycerol-3-phosphate dehydrogenase 1-like protein, GPD1L	gi|154152135	6.13;	38841	42 %	133	10
18	1933	Down	complement component 4 binding protein, alpha chain-like, C4BP	gi|76677514	6.34;	22393	42 %	123	6
19	1949	Down	complement component C9 precursor	gi|78369352	5.66;	63327	18 %	101	7

### Function enrichment and GO analysis of DEP

**Fig. 3:**
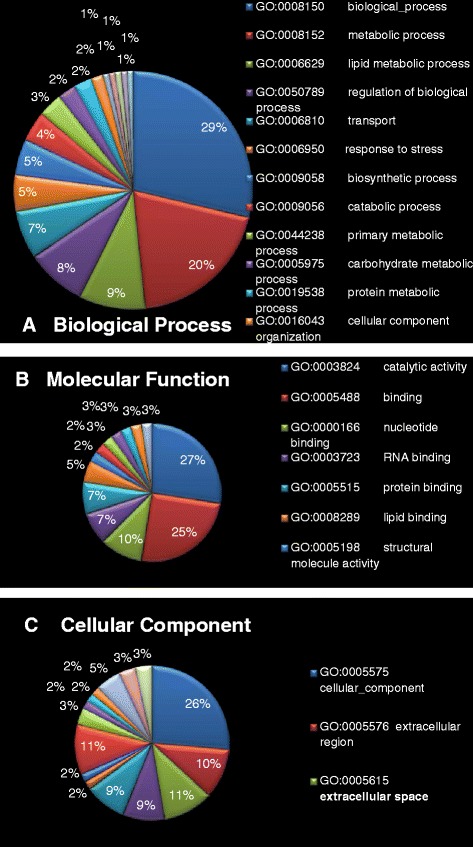
The GO analysis of differently expressed protein. **a** is biological process; (**b**) is molecular function and (**c**) is cellular component

To gain a better understanding of the 16 differentially expressed proteins identified in this study, further analysis was performed using bioinformatics. The identified proteins were categorized according to their function based on published literature and Uniprot database. These differential proteins were mainly classified by function into the following categories: carbohydrate metabolism, lipid metabolism, molecular transporter, immune regulation, inflammatory reaction, oxidative stress, and so on. By the GO analysis of AgBase, BPs of DEPs were mainly classified as biological processs (29 %), metabolic process (20 %), lipid metabolic process (9 %), regulation of biological process (8 %), transport of biomolecules (7 %), response to stress (5 %), and so on (Fig. 3a). MFs of DEPs were mainly classified as catalytic activity (27 %), binding (25 %), nucleotide binding (10 %), RNA binding (7 %), protein binding (7 %), lipid binding (5 %), and so on (Fig. 3b). CCs of DEPs were mainly classified as cellular component (26 %), cytoplasm (11 %), extracellular region (11 %), extracellular space (11 %), intracellular region (9 %), cell (9 %), and so on (Fig. 3c).

### Validation of 2-DE results

**Fig. 4:**
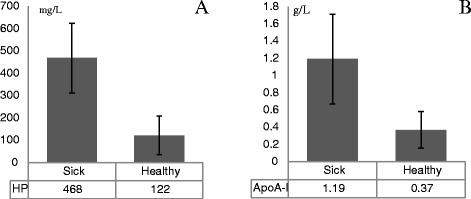
Concentration of HP (**a**) and ApoA-I (**b**) of plasma by ELISA in the sick and healthy cows. Note. All data are expressed as mean ± SD, *p < 0.05*

To validate the accuracy of 2-DE results, haptoglobin and ApoA-I in plasma were successfully measured, respectively, by ELISA and immunoturbidimetric method. It indicated that the Mindray human test kits for ApoA-I was also suitable for bovine. The results showed that haptoglobin (*P* < 0.01) and ApoA-I (*P* < 0.05) in the cows with laminitis were significantly higher than those in the healthy cows (Fig. 4). The differences in haptoglobin and ApoA-I concentrations were confirmed again. Although the fold changes were different from the results by 2-DE, the changing trend was consistent with2-DE, enhancing the possibility of the data of 2-DE being reliable.

### Analysis of antioxidant ability of plasma in dairy cows

There were significant differences in the concentrations of T-AOC, MDA, and GSH-Px between sick and healthy groups (*P* < 0.01), but the concentration of SOD was not different between them (Table 2). The results indicated that the antioxidant ability of plasma decreased and the redox equilibrium was disturbed—caused by oxidative stress—in the sick cows. It was consistent with the proteomic results.

**Table 2:** Antioxidant ability of plasma in dairy cows

Group	Number	T-AOC	SOD	MDA	GSH-Px
		umol/L	U/mL	nmol/mL	U/mL
Sick	36	5.82 ± 0.2a	58 ± 7.2a	3.4 ± 0.5a	27 ± 3.4a
Health	15	7.2 ± 1.45b	64 ± 4.6a	2.2 ± 0.1b	41 ± 4.2b

Different letters in the same column mean significant difference (*P* < 0.01)

## Discussion

Laminitis is a major cause of lameness in dairy cattle, which leads to serious economic loss for producers. But so far, the etiology and pathogenesis of laminitis in cow are not clear. The current situation gives us so many difficulties to prevent the disease. Comparative proteomics provide a novel pathway to resolve the question, Hannah [19] analyzed the proteomics of lamellar tissue of equine laminitis, and the results showed that COMP and keratins could serve as serum biomarkers for the developmental phase of laminitis. This study applied proteomics technology to compare the difference in the expression of plasma proteome between healthy and laminitis cows. A total of 16 DE proteins were discovered of which 4 were down-regulated and 12 were up-regulated in expression. These DE proteins were involved in the pathways such as carbohydrate metabolism, lipids metabolism, molecular transport, immune regulation, inflammatory reaction, oxidative stress, and so on. The result has laid a good foundation to further understand the pathogenesis of laminitis in dairy cow.

### HMGCR—up-regulated expression

HMGCR, transmembrane glycoprotein, is the rate-limiting enzyme in cholesterol biosynthesis [28]. The up-regulated expression of HMGCR leads to increase in the biosynthesis volume of cholesterol. In hyperlipidemia and atherosclerosis, the HMGCR activity usually increases, so the competitive inhibitors of the reductase (e.g., statins) are used to lower HMGCR. In laminitis of cows, HMGCR was overexpressed, resulting in cholesterol accumulation in vascular inner wall, especially in the microcirculation blood capillary in hoof corium. The accumulation may cause blood hypoperfusion, even arteriosclerosis [29, 30], and then the occurrence of laminitis. The conclusion could be in accordance with the previous result [1]. So the lipid metabolic disturbance induced by the up-regulated expression of HMGR may be a pathway to cause laminitis in dairy cattle.

### ApoA-I and ApoA-IV—up-regulated expression

Apolipoprotein is constituted of an important component of plasma lipoproteins, which have been found to bind lipopolysaccharide (LPS) *in vitro* and neutralize its toxic effects [31]. *In vitro*, apoA-IV plays anti-inflammatory and antiatherogenic roles after the administration of LPS [32, 33]. Therefore, apolipoprotein may play a vital role in perpetuating the inflammatory response. In addition, ApoA-I participates in the reverse transport of cholesterol from tissues to the liver for excretion by promoting cholesterol efflux from tissues and by acting as a cofactor for the lecithin cholesterol acyltransferase. ApoA-IV is a major component of high-density lipoprotein, which can promote cholesterol efflux to an equal extent from adipose cells [34]. In bovine, subacute ruminal acidosis was considered as the main cause of laminitis with the high level of LPS in blood [3, 12]. Therefore, ApoA-I and ApoA-IV were both up-regulated in expression to enhance the ability of the organism to neutralize the LPS toxicity; these proteins can transport cholesterol from tissue to liver for catabolism. In a research on horse with chronic laminitis, ApoA-IV was also found up-regulated in expression to play an anti-inflammatory role [35], which was in accordance with the findings of this study.

### Haptoglobin—up-regulated expression

Haptoglobin is an important acute-phase protein, which can assess the innate immune system’s systemic response to infection, inflammation, or trauma, including hemoglobin-binding capacity, in maintaining the iron homeostasis in cattle [36]. Haptoglobin also functions as a bacteriostatic agent, which increases rapidly during an infectious disease. At acute clinical mastitis in dairy cows, the haptoglobin concentration increases markedly both in blood and milk [37]. Haptoglobin concentrations increase to sixfold in dairy cows with infectious and metabolic diseases at slaughter compared to animals with minor lesions [38], In cattle, haptoglobin is effective in the diagnosis and prognosis of mastitis, enteritis, peritonitis, pneumonia, endocarditis, and endometritis [39]. Elevations in this protein have also been reported in cows with fatty liver syndrome, at parturition and during periods of starvation and transport stress, but not in laminitis. In this study, haptoglobin expression was found to be up-regulated in the case of laminitis, which prevents loss of iron and plays a vital role in anti-inflammatory reaction.

### Conglutinin—up-regulated expression

Conglutinin is a 371-amino acid calcium-dependent serum lectin specific for N-acetylglucosamine and plays an important role in defense mechanisms [40]. Conglutinin of cow is a member of the collectin protein family, which is an effector molecule in innate, non-adaptive immune defense against microorganism pathogens. Conglutinin binds to microbial surface wall and immune complexes through the complement component (C3bi), thereby impeding infectivity or mediating phagocytosis through specific receptors on phagocytes [41]. In the development of laminitis, conglutinin was up-regulated in expression, which indicated that the natural immune ability of cows may be improved.

## Conclusions

The current study provides a novel report on plasma proteomic profiles of laminitis in dairy cows. The results highlight the differentially expressed proteins in blood plasma between healthy dairy cows and clinical laminitis cows, including 12 proteins up-regulated and 4 proteins down-regulated. Overall, these differential proteins were involved in the pathways including carbohydrate metabolism, lipid metabolism, molecular transport, immune regulation, inflammatory reaction, oxidative stress, and so on. The differentially expressed proteins were related to the occurrence and development of laminitis and the lipid metabolic disturbance may be a new pathway to cause laminitis in dairy cows. The results may provide some clues for further revealing the pathogenesis of laminitis and screening the early diagnostic proteins and therapeutic drug target.

## Abbreviations

BP: Biological process
CAAS: Chinese academy of agricultural sciences
CC: Cellular component
CHAPS: 3-[(3-Cholamidopropyl) dimethylammonio]-1-propanesulfonate
DEP: Differentially expressed proteins
DTT: Dithiothreitol
EDTA-K2: Dipotassium ethylene diamine tetra -acetate
ELISA: Enzyme-linked immunosorbent assay
GO: Gene ontology
GSH-Px: Glutathione peroxidase
IAA: Iodoacetamide
IEF: Isoelectric Focusing
IPG: Immobilized pH gradient
MALDI-TOF MS: Matrix-assisted laser desorption/ionization time-of-flight mass spectrometry
McAb: Monoclonal antibody
MDA: Malonaldehyde
MF: Molecular function
PBST: Phosphate buffer solution and Tween-20
PMF: Peptide mass fingerprinting
SOD: Super oxygen dehydrogenases
T-AOC: Total antioxidative capacity
2-D: Two-dimensional gel
2-DE: Two-dimensional gel electrophoresis
